# Improved water purification by PVDF ultrafiltration membrane modified with GO-PVA-NaAlg hydrogel

**DOI:** 10.1038/s41598-023-35027-5

**Published:** 2023-05-18

**Authors:** Armin Ghobadi Moghadam, Alireza Hemmati

**Affiliations:** School of Chemical, Petroleum and Gas Engineering, Iran University of Science and Technology, Tehran, Iran

**Keywords:** Environmental sciences, Materials science, Nanoscale materials

## Abstract

This work presents a modified polyvinylidene fluoride (PVDF) ultrafiltration membrane blended with graphene oxide-polyvinyl alcohol-sodium alginate (GO-PVA-NaAlg) hydrogel (HG) and polyvinylpyrrolidone (PVP) prepared by the immersion precipitation induced phase inversion approach. Characteristics of the membranes with different HG and PVP concentrations were analyzed by field emission scanning electron microscopy (FESEM), Atomic force microscopy (AFM), contact angle measurement (CA), and Attenuated total reflectance Fourier transform infrared spectroscopy (ATR-FTIR). The FESEM images showed an asymmetric structure of the fabricated membranes, and possessing a thin dense layer over the top and a layer finger-like. With increasing HG content, membrane surface roughness increases so that highest surface roughness for the membrane containing 1wt% HG is with a Ra value of 281.4 nm. Also, the contact angle of the membrane reaches from 82.5° in bare PVDF membrane to 65.1° in the membrane containing 1wt% HG. The influences of adding HG and PVP to the casting solution on pure water flux (PWF), hydrophilicity, anti-fouling ability, and dye rejection efficiency were evaluated. The highest water flux reached 103.2 L/m^2^ h at 3 bar for the modified PVDF membranes containing 0.3 wt% HG and 1.0wt% PVP. This membrane exhibited a rejection efficiency of higher than 92%, 95%, and 98% for Methyl Orange (MO), Conge Red (CR), and Bovine Serum Albumin (BSA), respectively. All nanocomposite membranes possessed a flux recovery ratio (FRR) higher than bare PVDF membranes, and the best anti-fouling performance of 90.1% was relevant to the membrane containing 0.3 wt% HG. The improved filtration performance of the HG-modified membranes was due to the enhanced hydrophilicity, porosity, mean pore size, and surface roughness after introducing HG.

## Introduction

Water is a vital living source that organisms need to survive; however, only a limited amount of available water contains freshwater^1,2^. One of the major pollutants in wastewater released from various industries, mainly textiles and paper, are dyes that cause health risks to humans and create detrimental environmental problems^3^. Dyes from the textile industry have high molecular weight, non-biodegradability, toxic reactants, and complex structures. Moreover, dyes as a barrier to light emission disrupt the aquatic plants' growth; therefore, treating such effluents is vital^4,5^. Common methods for treating dye-contained wastewater include chemical/photocatalytic oxidation, adsorption, membrane separation, and coagulation^6^. Due to the high energy requirement and limited reusability of conventional treatment technologies, membrane separation processes with high efficiency, ease of operation, and reduced energy consumption have received more attention^7,8^. Membrane processes based on pressure force include microfiltration (MF)^9,10^, ultrafiltration (UF)^11^, nanofiltration (NF)^12^, and reverse osmosis (RO)^13^. The UF technique is broadly employed in treating water polluted with different dyes^14^.

Polyvinylidene fluoride (PVDF) is a suitable substance for manufacturing UF polymeric membranes owing to its good chemical resistance, thermal stability, and mechanical strength^15^. PVDF membranes are relatively hydrophobic or less hydrophilic, thereby causing membrane fouling by proteins and organic matters throughout wastewater treatment. Membrane fouling reduces its lifespan and water flux while increases energy costs^16^. Hence, membrane hydrophilicity is significantly decreased^9^. Various techniques can improve hydrophilicity and anti-fouling characteristics of such membranes; for example, inorganic nanoparticles have been incorporated into membrane matrix, resulting in increased hydrophilicity and anti-fouling. Wu et al. synthesized the PVDF-SiO_2_ composite membrane via the phase inversion (PI) approach, exhibiting that adding SiO_2_ improved the membrane fouling resistance^17^. Yan et al. synthesized modified PVDF/Al_2_O_3_ membranes via the PI method. AFM and SEM analyses demonstrated that Al_2_O_3_ nanoparticles enhanced the membrane anti-fouling performance and permeation flux compared to the unmodified membrane^18^. Other nanoparticles include titanium dioxide (TiO_2_)^19^, ferroferric oxide (Fe_3_O_4_)^20^, carbon nanotubes (CNTs)^21^, and graphene oxide (GO)^22^, which have been utilized in membrane fabrication to remove dyes successfully.

GO is a nano-scale derivative of carbon-based materials, which has attracted much consideration in preparing nanocomposite membranes because of its unique features, such as extensive specific surface area, proper hydrophilic nature, high strength, thermal stability, and chemical inertia^23^. In addition, it is a two-dimensional (2D) substance made in an individual layer with a hexagonal architecture having functional groups containing oxygen, like hydroxyl, carboxyl, epoxy, and carbonyl, located on the basal planes and edges^24^. Beygmohammdi et al. embedded GO nanosheets into PVDF membranes, and the results indicated an improved anti-fouling property of PVDF membranes caused by the hydrophilicity of GO^25^. Zhao et al. fabricated PVDF/GO ultrafiltration membranes via the PI procedure, showing enhanced hydrophilicity, water flux, and anti-fouling performance^26^.

Polyvinyl alcohol (PVA) is a polymer with unique features, such as mechanical and thermal stability, biocompatibility, film formation capability, hydrophilic nature and chemical stability. Since PVA polymer is water soluble, cross-linking of PVA is essential for applications in contact with water or aqueous solutions^27^. Yoon et al. prepared PVA hydrogel membranes by cross-linking with sulfosuccinic acid (SSA). Their observations illustrated that the fabricated membranes had good mechanical properties, regeneration capability, excellent reusability, and high adsorption capacity^28^. PVA composite membranes functionalized with d-glucose and agar were prepared by Nguyen et al.^29^ via the soluble casting method for dye-contaminated water treatment. They reported that the thermal stability, hydrophobicity, and mechanical strength of the composite membranes advanced because of the presence of PVA. Today, the use of polymer hydrogel membranes in wastewater treatment has been broadly considered^30^.

Sodium alginate (NaAlg) is a natural biopolymer made from seaweed, which is a cheap, efficient material, particularly for treating water because of its supreme inherent characteristics, particularly environmental friendliness and non-toxicity^31^. Amiri et al. prepared polyethersulfone (PES)/(PVAGO-NaAlg) hydrogel nanocomposite membranes via PI approach for water treatment. The membrane anti-fouling, permeability, and dye rejection increased when increasing the hydrogel concentration to 1 wt% as compared with the neat membranes^32^.

Polyvinylpyrrolidone (PVP) is another polymeric material used to modify PVDF polymer membranes. It is a pore-forming agent in such membranes because of its good hydrophilicity^33^. Ahmadzadeh et al. made PVDF nanocomposite membranes with GO nanoribbons and PVP by the phase inversion method. They demonstrated that the water flux of PVDF/PVP membrane was enhanced by 80% compared with the unmodified PVDF membrane and the improved anti-fouling properties^34^.

Table 1 summarizes the PVDF-based nanocomposite membranes for removing dye contaminants from water. As reported, using organic and inorganic nanomaterials in PVDF membranes could advance the hydrophilic features of the membrane and efficiently eliminate various dyes. The objective of this study is to develop PVDF membrane using nanocomposite hydrogel with the aim to improve permeability and antifouling properties. In this work, GO-PVA-NaAlg nanocomposite hydrogel was cast with PVDF to prepare nanocomposite UF membranes to increase the resistance to membrane foulant and enhance dye removal efficiency. Conge red (CR), methyl orange (MO) dyes as well as bovine serum albumin (BSA) were utilized to evaluate the membrane performance in a dead-end filtration setup. The effects of adding different compositions of PVA-GO-NaAlg hydrogel and PVP on morphology, hydrophilicity, and performance of fabricated UF membranes were assessed. Moreover, the anti-fouling characteristics of UF membranes were analyzed by BSA solution filtration.

**Table 1:** A summary of recent nanocomposite membranes for wastewater treatment.

Nanocomposite membrane	Membrane type	Rejection (%)		Flux (L/m^2^h)	Remarks	References
PVDF/PANI*/GO	UF	Allura Red 98%	Methyl Orange 95%	454	High-flux, dye separation and antifouling	^14^
PVDF/HDTMA/clinoptilolite	NF	Reactive Red 120 98.5%		18	Dye separation	^35^
PVDF/PVP/Fe_3_O_4_-HNTs	NF	Methylene Blue 84.7%	Conge Red 92.1%	39.8	Dye separation and antifouling	^12^
PVDF/PVP/GO-ZnO	UF	BSA 93%		170.7	BSA separation and antifouling	^36^
PVDF/PVP/D-A-HNTs	UF	Direct Red 28 86.5%	Direct Yellow 4 85%	42.2	Dye separation and antifouling	^37^
		Direct Blue 14 93.7%				
PVDF/PVP/GONRs	UF	BSA 95%		532.3	High-flux, BSA separation and antifouling	^34^
PVDF/PVP/Cu_2_S	UF	Reactive Blue 21 98.2–99.5	Direct Black 38 96.2–98.1	248.25	Dye separation	^38^
		Direct Yellow 12 50.3/64.7				
PVDF/PEG/ZnOPb	UF	Reactive Black 5 98.9%		–	Dye separation	^39^
PVDF/PVP/GO	UF	BSA 87%		505	BSA separation and antifouling	^26^
PVDF/PVP/GO-PVA-NaAlg	UF	Conge Red	Methyl Orange	–	–	This work

*PANI* polyaniline, *HDTMA* hexadecyltrimethylammonium bromide, *HNTs* halloysite nanotubes, *GONRs* graphene oxide nanoribbones.

## Experimental

### Materials

PVDF powder (Kynar 761) was supplied by Arkema France and utilized as the polymer matrix. N,N-dimethylacetamide (DMAc) solvent (99%), BSA (67,000 g/mol), PVP (58,000 g/mol) and PVA (85,000 g/mol) were purchased from Merck Co. (Germany). Graphite powder, phosphorous pentoxide (P_2_O_5_), potassium persulfate (K_2_S_2_O_8_), sulfuric acid (H_2_SO_4_, 98%), hydrogen peroxide (H_2_O_2_, 30%), sodium alginate, boric acid (H_3_BO_3_) and calcium chloride (CaCl_2_), potassium permanganate (KMnO_4_, 99%) and hydrochloric acid (HCl, 37%) were purchased from Sigma Aldrich (USA) and harnessed to synthesize GO and GO-PVA-NaAlg. Methyl orange (327.33 g/mol) and Conge red (696.66 g/mol) anionic Dyes were used to make synthetic effluent.

### Synthesis of GO nanosheet

GO was synthesized via the modified Hummer’s approach^40^. Briefly, 1.50 g of graphite powder, 1.25 g of P_2_O_5_, and 1.25 g of K_2_S_2_O_8_ were dispersed in 20 mL of H_2_SO_4_ upon a magnetic stirrer at 80 °C for 6 h. Next, 500 mL of deionized (DI) water was poured slowly after cooling the solution to ambient temperature, and the solution was vacuum-filtrated. The DI water was used to wash the pre-oxidized GO, then it was dried at room temperature, and dispersed in 60 mL of H_2_SO_4_. Then, 4.5 g of KMnO_4_ was added to the solution, the resultant mixture was stirred at 35 °C for 2 h, followed by 1000 mL of DI water and 7.5 mL of H_2_O_2_ slowly poured into the solution. After 24 h, the solution precipitated, and the precipitated residual was washed with HCl (30 wt%) and DI water using centrifugation. Finally, the resulting GO was dried at 70 °C and stored for following use.

### Synthesis of GO-PVA-NaAlg

The preparation steps of GO-PVA-NaAlg granules are as follows.

1. 0.5 g of GO in DI water (50 mL) was sonicated during 1 h at room temperature.
2. 1.25 g of NaAlg was dropped and sonicated for 1 h to disperse the solution.
3. 0.5 g of PVA was poured into the solution under mixing and heating up to 90 °C to complete the dissolution.

After natural cooling, saturated H_3_BO_3_/CaCl_2_ solution (4.0 wt%) was poured into the mixture to obtain nanocomposite hydrogel beads. Ultimately, the formed beads were washed with distilled water, dried with a freeze dryer, and powdered to form UF membranes^24,32^.

### Synthesis of PVDF membrane blended with GO-PVA-NaAlg

Nanocomposite hydrogel membranes were synthesized via PI approach^35,41^. To prepare the membrane, first, specific amounts of nanocomposite hydrogels (0.1, 0.3, 0.5 and 1.0 wt%) were added to a covered beaker and dispersed in DMAc solvent for 30 min in an ultrasonic bath. After sonicated, PVP was added and stirred for 30 min. PVDF polymer was poured into the mixed solvent, hydrogel, and PVP under continuous stirring at ambient temperature for 24 h until complete dissolution. Finally, the resulting solution remained in a stagnant environment for 24 h without stirring. Then, the solutions were poured onto a plate (glassy), and a thin layer with 175 μm thickness was applied using a hand-held casting knife and instantly immersed within a coagulation bath including distilled water at ambient temperature. After PI, the resulting membrane was kept in DI water to eliminate residuals within the PVDF/GO-PVA-NaAlg nanocomposite membrane. The detailed composition of all components in preparing membranes and the assigned codes are given in Table 2.

**Table 2:** The detailed composition of the casting solutions for fabricating nanocomposite membranes.

Code	Membrane	PVDF (wt%)	PVP (wt%)	GO-PVA-NaAlg (wt%)	DMAc (wt%)
M1	PVDF 18%	18.0	–	–	82.0
M2	PVP 1%	18.0	1.0	–	81.0
M3	PVA-GO-NaAlg 0.1%	18.0	1.0	0.1	80.9
M4	PVA-GO-NaAlg 0.3%	18.0	1.0	0.3	80.7
M5	PVA-GO-NaAlg 0.5%	18.0	1.0	0.5	80.5
M6	PVA-GO-NaAlg 1.0%	18.0	1.0	1.0	80.0

### Characterization of GO-PVA-NaAlg composite

A scanning electron microscope (FESEM, TeScan—Mira III, Czech Republic) was utilized to examine the artichecture and surface morphology of the GO and PVA-GO-NaAlg nanocomposite. Their functional groups were analyzed by Fourier-transform infrared spectroscopy (FTIR, PerkinElmer-Spectrum RXI, USA) analysis in 500–4000 cm^−1^. Powder X-ray diffraction (XRD) (Bourevestnik-DRON8, Russia) analysis was performed to analyze the crystalline structures of the synthesized samples.

### Membrane characterization

FESEM images of the membranes were examined to check their surface and cross-sectional morphology. The cross-sectional samples were broken with liquid N_2_ and then coated with an ultrathin gold layer to enhance electrical conductivity. The surface roughness was also checked by Atomic Force Microscopy (AFM, FemtoScan, Russia). All samples with an area of 10 × 10 μm^2^ were scanned. The functional groups of the membranes were evaluated by Attenuated total reflectance Fourier transform infrared spectroscopy (ATR-FTIR). Surface hydrophilicity evaluation was conducted by static water contact angles taken by a telescopic goniometer (AM7915MZT, Dino-lite, Taiwan) at 25 °C. The images were provided from three drops (3 mL) of DI water on the surface. The average of four measurements was contemplated as the contact angle quantity to increase the measurement accuracy. The overall porosity ($\varepsilon$) was computed according to the gravimetric approach, as calculated by Eq. (1). Every membranes was immersed within DI water for one day at ambient temperature^42^.

1 $$ \varepsilon = \frac{{w_{1} - w_{2} }}{\rho Al} $$

where *w*_*1*_ and *w*_*2*_ [kg] are the mass of wet and dry membranes, respectively, the term *l* is the thickness [m], *A* is the effective surface area [m^2^], and *ρ* is the DI density [kg/m^3^]. The mean pore radius (*r*_*m*_) is specified by measuring the water flux and porosity according to the Guerout-Elford-Ferry correlation, as given below^43^.

2 $$ r_{m} = \frac{{\sqrt {(2.9 - 1.75\varepsilon )8\eta lQ} }}{\varepsilon A\Delta P} $$

in which, *η* is the viscosity of water [Pa s], *Q* is the volume flow rate [m^3^/s], and *ΔP* is transmembrane pressure [Pa].

### Antifouling property

BSA protein was harnessed as a foulant on the nanocomposite membranes to test the check their anti-fouling. First, water flux (*J*_*W,1*_) was measured for 1 h. The BSA aqueous solution (100 mg/L) at a pH of 7 was measured for 1 h (*J*_*p*_). Next, the membrane was immersed in distilled water for 30 min to wash reversible fouling of the membranes. Finally, The second water flux (*J*_*W,2*_) was measured at the same condition of temperature and pressure. The membranes were assessed with the parameters of flux recovery ratio (*FRR*), total fouling ratio (*R*_*t*_), reversible fouling ratio (*R*_*r*_), and irreversible fouling ratio (*R*_*ir*_). These parameters were calculated by the equations below^44^:

3 $$ \% FRR = \frac{{J_{w,2} }}{{J_{w,1} }} \times 100 $$

4 $$ R_{t} = \frac{{J_{w,1} - J_{P} }}{{J_{w,1} }} \times 100 $$

5 $$ R_{r} = \frac{{J_{w,2} - J_{P} }}{{J_{w,1} }} \times 100 $$

6 $$ R_{ir} = \frac{{J_{w,1} - J_{w,2} }}{{J_{w,1} }} \times 100 $$

### Water flux

**Figure 1:**
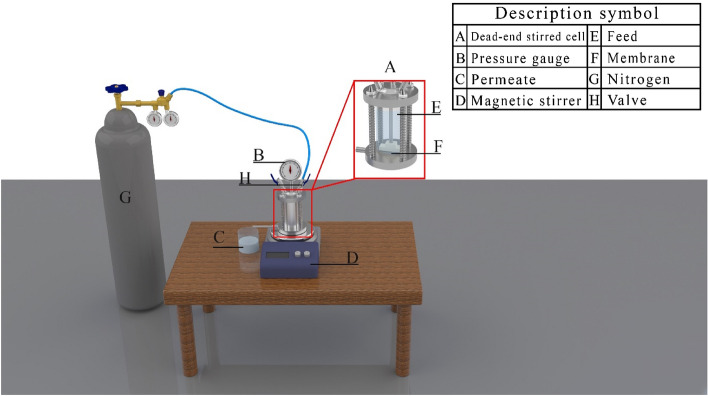
Scheme of the experimental dead-end filtration setup used in this study.

Membrane permeability and anti-fouling characteristics were evaluated by the filtration system including a stirred cell (220 mL and 18.5 cm^2^ effective area). The membranes were first compressed by water at 4 bar during 30 min to achieve a steady-state flux. Then, PWF (L/m^2^ h) was measured at 3 bar pressure and ambient temperature, computed as follows^45,46^:

7 $$ J = \frac{V}{At} $$

in which, *V* is the total volume of the water permeated (L), *A* is the effective area (m^2^), and *t* is the filtration time (h). Figure 1 depicts the schematic view of the experimental dead-end filtration setup.

### Rejection rate

The permeate flux and rejection rate of BSA, conge red, and methyl orange solutions (100 mg/L) were calculated after measuring water flux for 60 min. BSA and dyes rejection (*R*) was determined as follows^47^:

8 $$ \% R = \left( {1 - \frac{{C_{P} }}{{C_{F} }}} \right) \times 100 $$

where *C*_*F*_ and *C*_*P*_ are the contaminant concentrations in feed and permeate sides, determined by UV–Vis spectrophotometer (Shimadzu UV-1800, Japan) at maximum wavelengths of 280, 464, and 492 nm for BSA, methyl orange, and conge red, respectively.

## Results and discussion

### Characterization of GO-PVA-NaAlg composite

**Figure 2:**
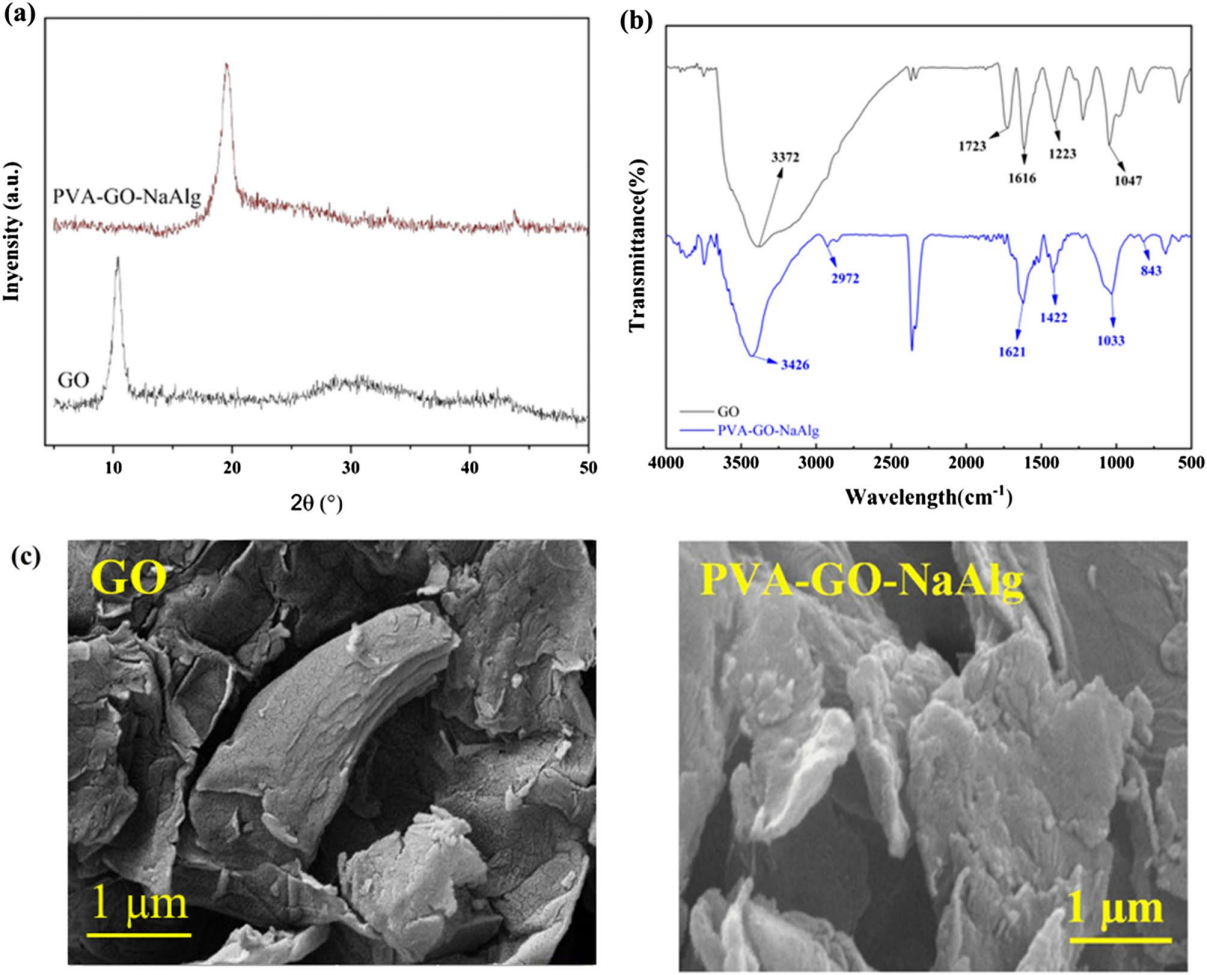
(**a**) XRD patterns, (**b**) FTIR spectra, and (**c**) FESEM images of GO nanosheets and GO-PVA-NaAlg.

The XRD patterns of GO nanosheet and GO-PVA-NaAlg composite in 10°–50° range are depicted in Fig. 2a. The diffraction peak at 10.39° can be assigned to the interlayer spacing of GO, obtaining an interlayer spacing of 0.85 nm. For GO-PVA-NaAlg nanocomposite, the interlayer spacing increased, but the (001) plane moved towards smaller angles. The diffraction peak at 19.5° can be associated with the creation of the crystalline form of PVA in GO nanosheets. Furthermore, other peaks at 31.1°, 33.1°, and 43.8° demonstrate the existence of calcium ions in NaAlg^48^.

GO-PVA-NaAlg hydrogel were i prepared n-situ through a solution intercalation procedure. Figure 2b compares the representative FTIR spectra of GO nanosheet and GO-PVA-NaAlg composite. For the GO spectrum, a robust peak at 3372 cm^−1^ corresponds to the hydroxyl (O–H) group stretching vibration. The characteristic bands at 1723 and 1616 cm^−1^ are associated with the stretching vibration of the carboxylic (C=O) group and the (C=C) group, respectively. The absorption bands at 1223 and 1047 cm^−1^ are assigned to the stretching vibration of (C–OH) and the epoxy (C–O–C) group, respectively^49,50^. For the nanocomposite sample, the bands at 3426 and 2922 cm^−1^ are related to O–H and –CH_2_– stretching vibrations. The bands at 1621 and 1422 cm^−1^ are respectively relevant to asymmetric and symmetric stretching vibrations of carboxylate ions (COO^−^). Also, the absorption bands at 1094 and 843 cm^−1^ are attributed to the stretching vibrations of C–OH and C–C bonds. Such results confirmed that NaAlg and PVA molecules exist in GO sheets via hydrogen bonding interactions^51^.

Figure 2c illustrates the FESEM images of the GO nanosheet and GO-PVA-NaAlg composite. The nanocomposite has a porous 3D structure as a combination of PVA and NaAlg molecules through the hydrogen bonding interactions within GO layers, resulting in the demolition of the orderly architecture of GO. The crosslinking of PVA/NaAlg with GO with the intercalated hydrogen bonding interactions has reduced the pore size and created a dense hydrogel network structure^51^.

### Characterization of composite membranes

**Figure 3:**
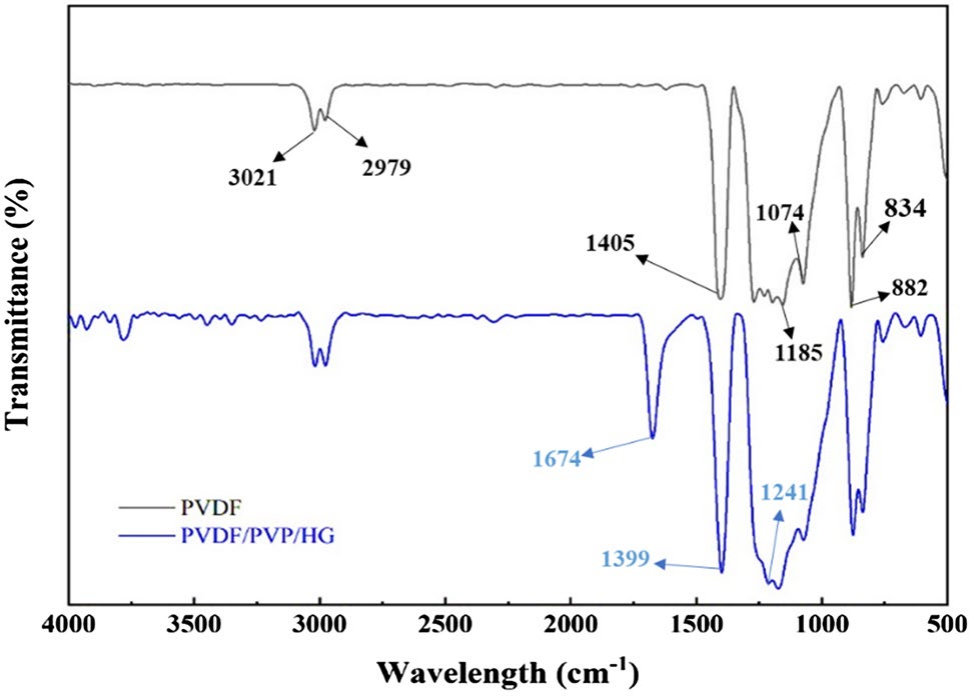
ATR-FTIR spectra of the unmodified and 0.3 wt% HG-modified PVDF membranes.

The FTIR spectra of the bare PVDF and nanocomposite membranes are presented in Fig. 3. For the bare PVDF membrane, the peaks observed at 3021 and 2979 cm^−1^ are associated with the asymmetric and symmetrical vibration of the CH_2_ functional groups. The bands at 882 and 838 cm^−1^ correspond to stretching vibrations of C–C–C and C–F bonds. The peaks at 1405 and 1185 cm^−1^ demonstrate the CH_2_ and C–C groups, respectively. The absorption peak at 1074 cm^−1^ is also attributed to the C–O group^52^. In PVDF/PVP/GO-PVA-NaAlg membrane’s spectrum, the peaks formed are located in almost the same position as bare PVDF peaks. This phenomenon can be explained because of the trace amount of HG with 0.3 wt% in the membrane matrix. However, the strong bands at 1674, 1399 and 1241 correspond to the stretching vibrations of C=O, C–H and C–N groups, respectively, which proves the presence of PVP in the composite membrane structure.

**Figure 4:**
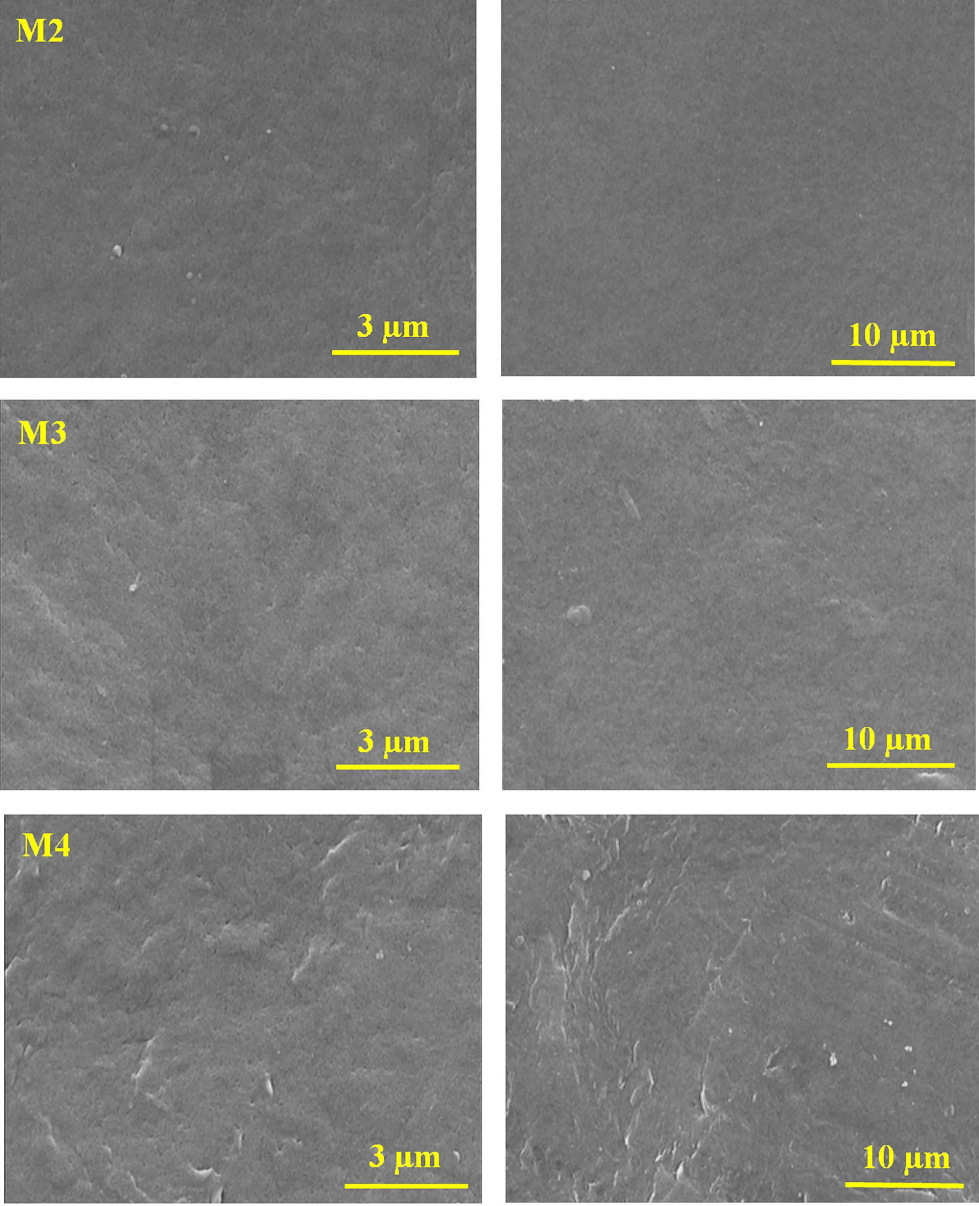

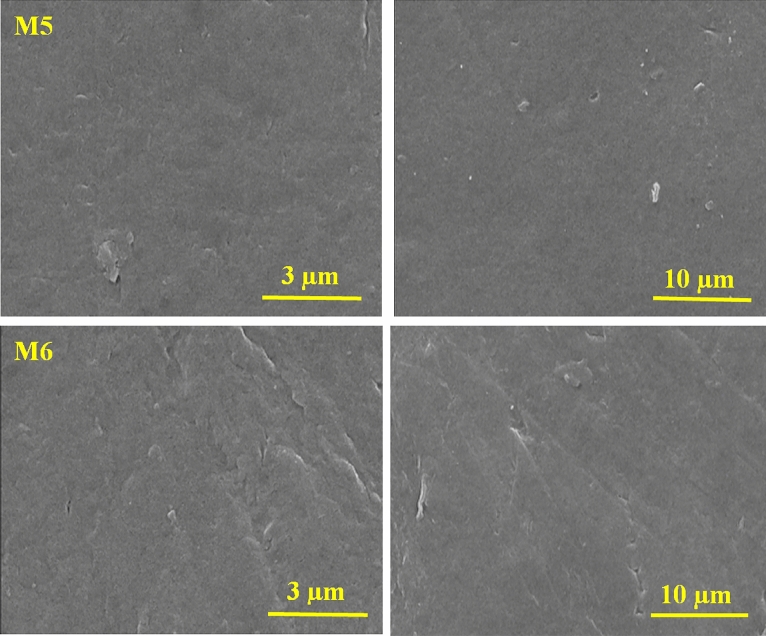
FESEM images of the bare PVDF (M1), PVDF/PVP (M2) and nanocomposite membranes (M3–M6).

The unmodified and HG-modified PVDF membranes were evaluated by scanning electron microscopy to examine the effect of adding hydrogel and PVP as the hydrophilic polymer on the membrane surface. Figure 4 depicts the FESEM images of membranes fabricated with different HG concentrations. As can be seen, by adding HG, no cracks are observed on the membranes’ surface, and it does not make the membrane brittle. The density of surface pores of the HG membrane increases compared with the bare PVDF one, in which the M4 membrane with 0.3 wt% HG/PVDF possessed the maximum pore numbers; however, the surface pore density in M5 and M6 membranes decreased, which can be related to the enhanced viscosity of polymeric solutions containing nanocomposite^35^.

**Figure 5:**
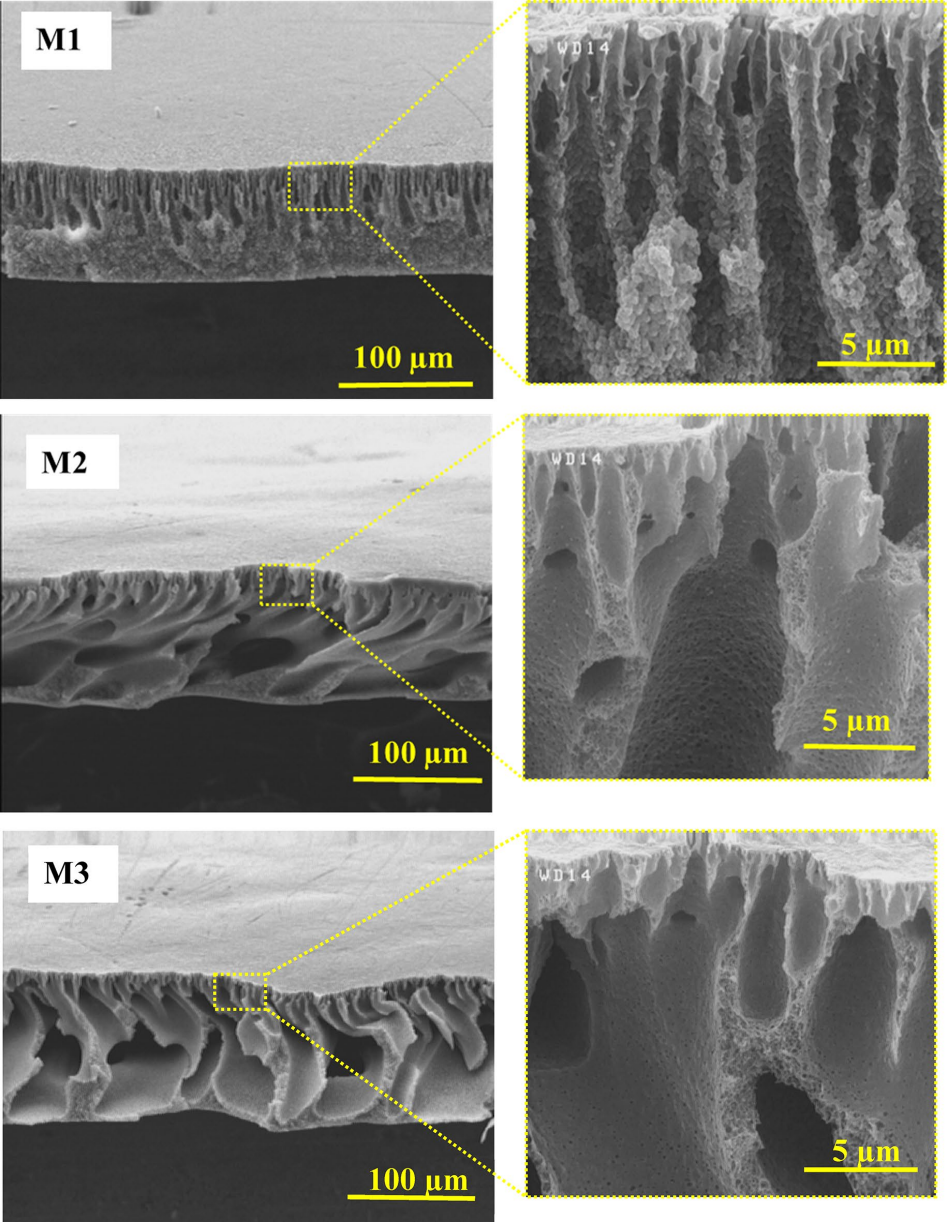

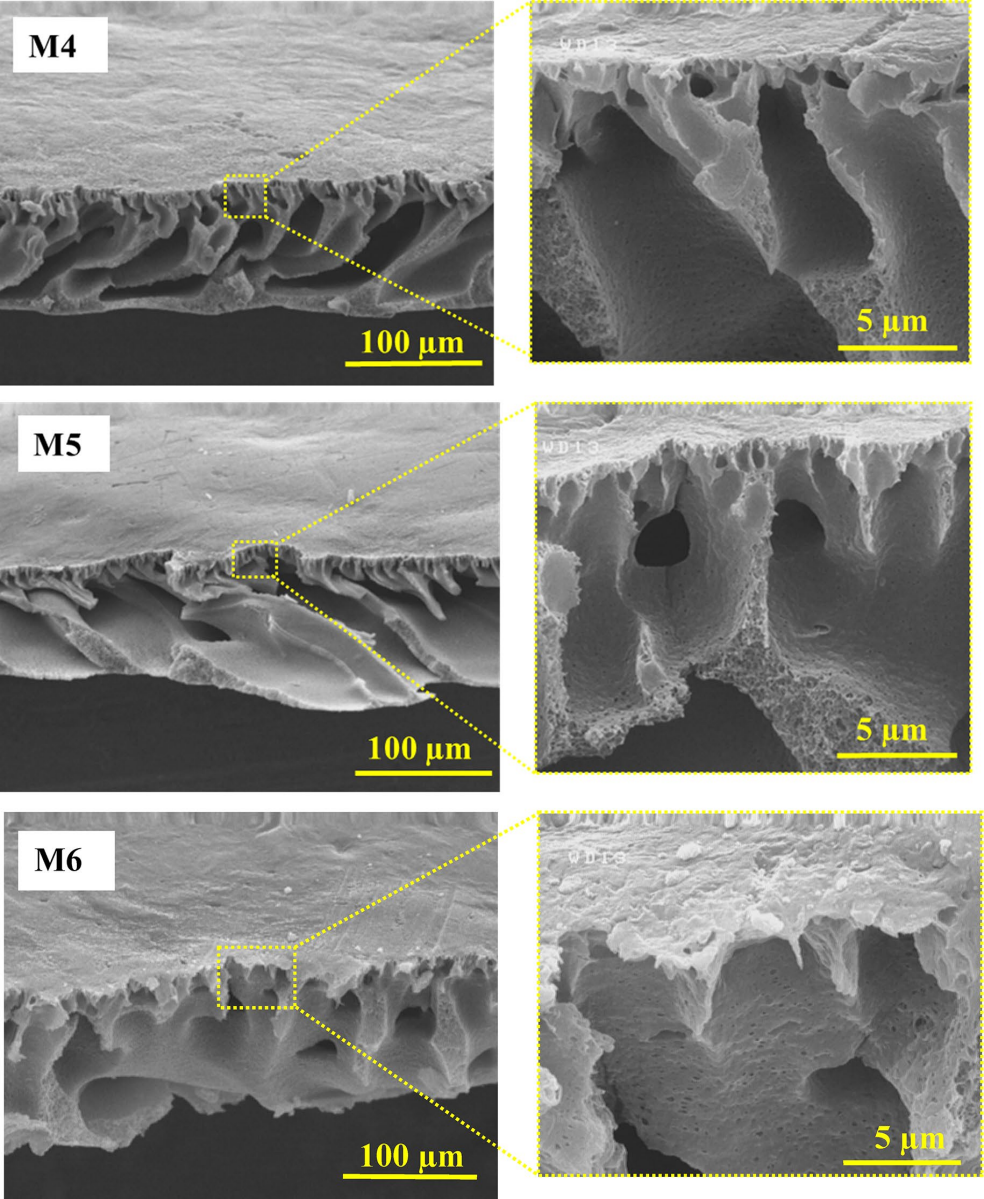
Cross-sectional FESEM images of the bare PVDF (M1), PVDF/PVP (M2) and nanocomposite hydrogel (M3–M6) membranes.

According to Fig. 5, the cross-sectional FESEM images exhibited an asymmetric structure of the fabricated membranes, and there is a thin dense layer over the top and a layer with a finger-like architecture in the nanocomposite membranes. Such a structure in membranes is because of the quicker PI process of PVDF within the coagulation bath. The PVDF/PVP membrane's porosity has increased with the number of random dispersed channels and height. Additionally, introducing HG nanocomposite into the PVDF membrane structure increased the finger-like pores, in which the largest pore size was detected for the 0.3 wt% HG membrane. The casting solution's viscosity increased with the HG fillers’ content. The excessive nanocomposite content, i.e., 0.5 and 1.0 wt%, decreases the number of pores, which can be associated with the accumulation of HG at higher concentrations^53,54^.

**Figure 6:**
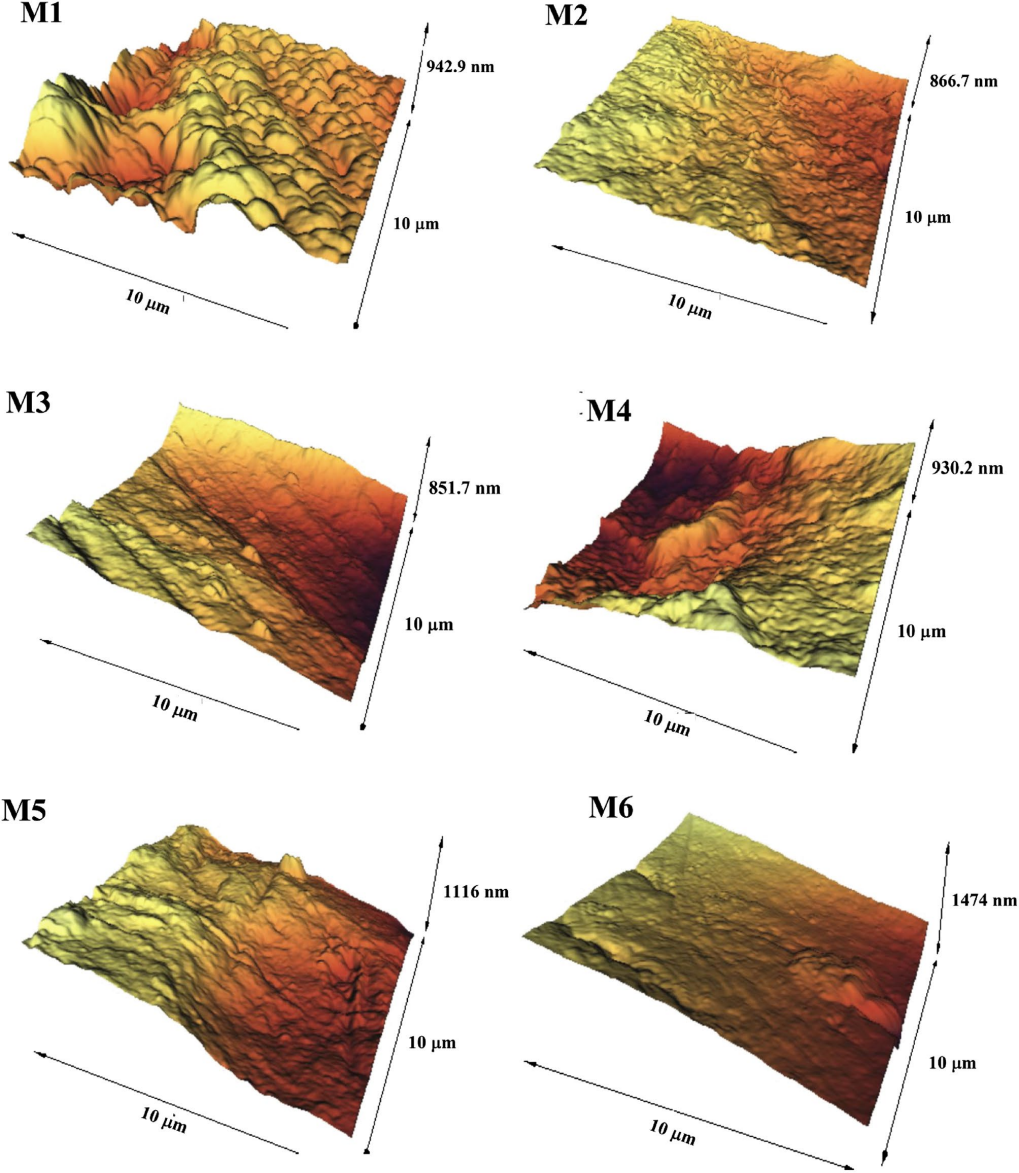
Surface AFM images of the bare PVDF (M1), PVDF/PVP (M2), and nanocomposite membranes (M3–M6).

Figure 6 illustrates the 3D surface obtained from AFM analysis to investigate the effect of GO-PVA-NaAlg hydrogels on the surface roughness of the PVDF membrane under a scan size of 10 μm × 10 μm. The brightest and darkest regions illustrate the peaks (highest section) and valleys (pores), respectively. Also, the mean surface roughness (*R*_*a*_) is reported in Table 3. The AFM images exhibit that the surface roughness of the membrane increases effectively by enhancing HG content. The *R*_*a*_ value is 128.9 nm in the bare PVDF membrane with strong hydrophobicity. The highest surface roughness for the membrane containing 1 wt% HG is with a *R*_*a*_ value of 281.4 nm. Adding HG-PVP to PVDF matrix enhanced the surface roughness, membrane hydrophilicity, and the solvent/non-solvent exchange rate throughout the PI process. Increasing the surface roughness in hydrophobic membranes diminishes their anti-fouling performance^34,43^.

**Table 3:** Roughness, contact angle, porosity, and mean pore size of all fabricated membranes.

Membrane	Roughness (nm)	Contact angle (°)	Porosity (%)	Mean pore radius (nm)
M1	128.9	82.5	70.8 ± 1.3	10.2
M2	152.2	76.9	73.0 ± 0.9	13.1
M3	189.3	73.1	75.7 ± 0.6	13.9
M4	233.9	70.7	80.5 ± 0.8	15.8
M5	245.6	68.4	79.0 ± 0.9	14.5
M6	281.4	65.1	77.8 ± 1.1	14.3

**Figure 7:**
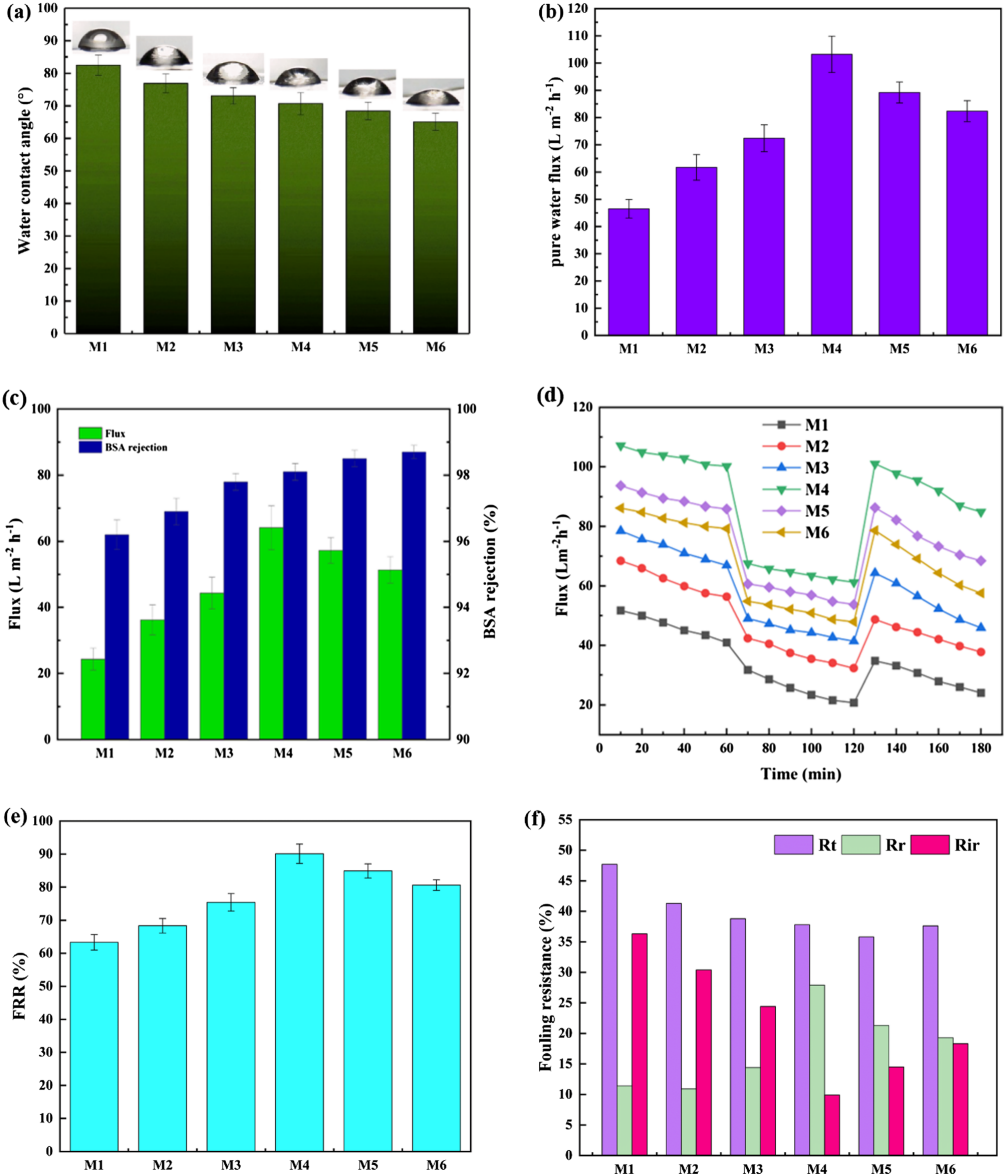
(**a**) Water contact angle; (**b**) Effect of hydrogel addition to membrane on water flux; (**c**) Permeation flux and rejection rate of BSA solution; (**d**) Flux versus time at 3 bar during three consecutive steps: water flux, flux of BSA solution (100 mg/L), and water flux after 20 min washing with distilled water; (**e**) Flux recovery; (**f**) Fouling resistances for nanocomposite membranes.

Figure 7a depicts the surface hydrophilicity of UF membranes containing the bare and mixed matrix PVDF by analyzing the water contact. The lower the contact angle, the higher the membrane hydrophilicity. The bare PVDF membranes exhibited the maximum contact angle with 82.5°^55^. Also, in PVDF/PVP membranes, the contact angle is about 76.9° due to the addition of PVP as a water-soluble polar substance that has raised the membrane hydrophilicity^26^. Adding PVP and GO-PVA-NaAlg hydrogel to the PVDF matrix reduced the contact angle, resulting in improved membrane hydrophilicity. The lowest contact angle of 65.1° was assigned to the membrane containing 1 wt% HG. The polar groups, such as hydroxyl, carboxyl, carboxylate ions and epoxy, in nanocomposites lowered the water contact angle, thereby improving the hydrophilic ability of PVDF/PVP/GO-PVA-NaAlg membranes^56^.

Table 3 reports the membranes’ porosity and average pore radius size determined by gravimetric test. The overall porosity was 70.8% for the bare PVDF membrane, whereas it reached 80.5% by adding PVA-GO-NaAlg nanocomposite, in which the highest porosity was obtained for the membrane M4 with 0.3 wt% HG and 1.0 wt% PVP. This pore size enlargement can be due to the hydrophilic inherent characteristic of HG and PVP, promoting the diffusion of polymer solvents and non-solvent (water) throughout the PI process^57^. However, the average pore size and overall porosity of M5 and M6 membranes with 2.0 and 5.0 wt% HG were reduced as a result of the enhanced solution viscosity and the pore blocking effect.

### Filtration experiments

#### Effect of HG content

Figure 7b exhibits the water flux of the fabricated bare PVDF, PVDF/PVP and nanocomposite membranes. As shown, the flux of the PVDF/PVP membrane is larger than that of the bare PVDF because PVP as the pore-former increases the porosity and the hydrophilicity^43^. The nanocomposite membranes showed water fluxes higher than the bare PVDF and PVDF/PVP membranes due to improved membrane hydrophilicity (Fig. 7a) and enhanced porosity, as reported in Table 3. The flux increased by enhancing the weight fraction of HG in the polymeric solution. The M4 membrane with 0.3% HG demonstrated the highest flux quantity of 103.2 L/m^2^ h, increasing by 121.9% as compared with the bare PVDF one. However, increasing the nanocomposite hydrogel enhances the pore blockage and reduces the porosity, reducing the water flux. The flux was reduced for the membranes comprising of 0.5 and 1.0 wt% HG to 89.2 and 82.3 L/m^2^ h, respectively^58^.

#### Antifouling and rejection rate

Two main reasons for reducing BSA flux in UF membranes are membrane fouling and concentration polarization. BSA fouling further reduces flux because the high stirring rate (400 rpm) reduced the concentration polarization adjacent to the membrane surface^59^. Figure 7c illustrates the BSA solution's rejection rate and flux through all the prepared membranes. As mentioned earlier, the addition of PVP and PVA-GO-NaAlg in the polymeric solution decreases the fouling while increases the hydrophilicity. All nanocomposite membranes have a higher flux and BSA rejection than bare PVDF and PVDF /PVP membranes. The modified membrane with 0.3wt% HG has the highest BSA flux, equal to 64.1 L/m^2^ h. Also, more HG content than the optimum one (0.3 wt%) in the casting solution has increased the BSA removal. The membrane with 1 wt% HG has the maximum BSA rejection efficiency of 98.7%, which can be owing to its high hydrophilicity and anti-fouling property.

Figure 7d depicts the 60-min fluxes related to the consecutive water and BSA solution permeation followed by the second water permeation after washing the membrane with distilled water. The BSA permeation flux after the water permeation exhibited a significant reduction in the permeability. This phenomenon is due to the adsorption of BSA on the membrane surface, improving the fouling rate. The PVDF membrane with 0.3 wt% HG illustrated the maximum water and BSA fluxes, while they are more susceptible to fouling because of the rapid flux reduction versus time^60^.

The rejected components of the BSA solution accumulated on the membrane surface and pores, and the fouling phenomenon reduced the flux; consequently, its fouling can be reduced using a hydrophilic additive within the membrane matrix. Figure 7e displays the FRR for the fabricated nanocomposite membranes. The nanocomposite membranes have higher FRR values than that of the unmodified PVDF one. The FRR quantity for the bare PVDF membrane was 63.3%, while it improved to 90.1% for the PVDF membrane containing 0.3 wt% HG^61^. The presence of hydrophilic HGs on the membrane surface forms a thin layer of water as a barrier to prevent the adsorption of precipitate (here BSA), confirming the improvement of higher antifouling characteristics in nanocomposite membranes^62^. The antifouling property improved by adding more HG into the PVDF membrane matrix. However, the FRR reached 84.9% and 80.6% for the PVDF membranes with 0.5 and 1.0 wt% HG, respectively. This reduction might be due to the accumulation of nanocomposites and the enhancement of roughness of the hydrophobic PVDF membranes, reducing their antifouling property. Accordingly, the GO-PVA-NaAlg hydrogel and PVP agent play a remarkable role in enhancing the anti-fouling ability.

The fouling resistance parameters of the fabricated membranes, including *R*_*t*_, *R*_*r*_, and *R*_*ir*_, are reported in Fig. 7f. The bare PVDF membrane, due to their hydrophobicity, has the maximum total resistance and irreversible fouling with 47.7% and 36.3%. Nevertheless, the PVDF membrane containing 0.5wt% HG has the minimum total fouling, i.e., 35.8%, among the prepared membranes. Also, all nanocomposite membranes had lower total and irreversible fouling resistances than the bare PVDF membrane because of the increased hydrophilicity and decreased contact angle after adding nanocomposite^42,63^.

#### Dye permeation flux and rejection rate

**Figure 8:**
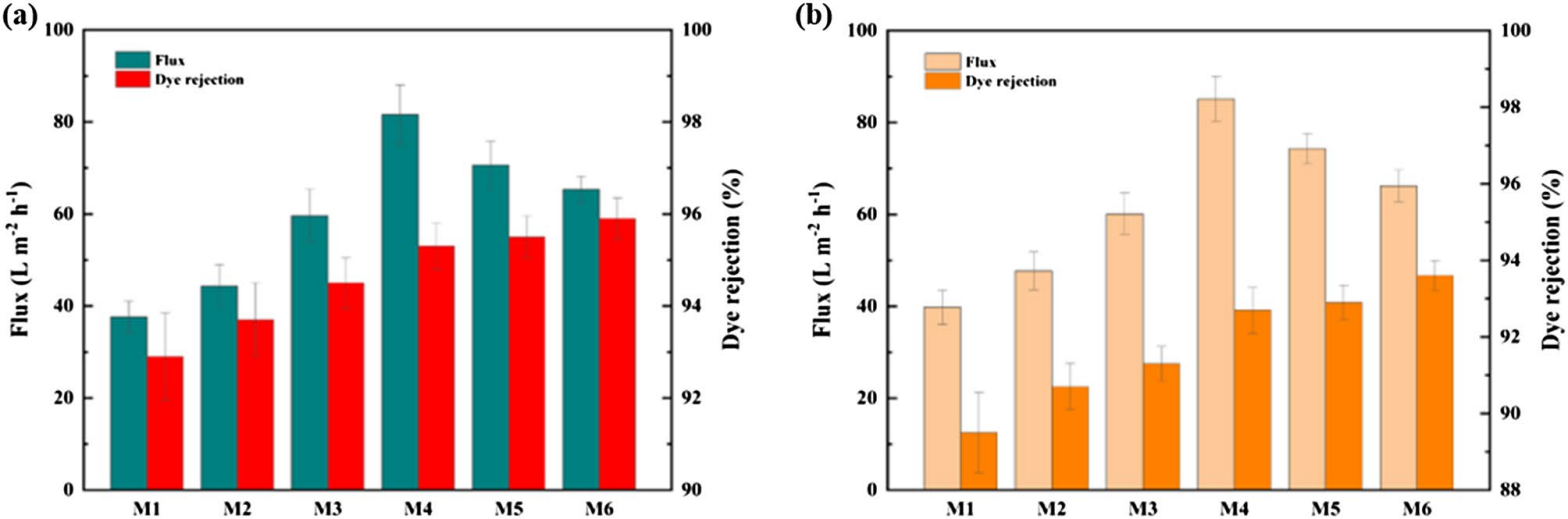
Permeation flux and rejection rate of (**a**) CR and (**b**) MO dyes by the bare PVDF membrane (M1), PVDF/PVP membrane (M2), and nanocomposite membranes (M3–M6).

Two dyes of Congo red (CR) and methyl orange (MO) were selected to assess the separation efficiencies of the synthesized membranes. The aqueous dyed solutions (100 mg/L) were filtrated with the membranes at 3 bar in the dead-end stirred cell. Figure 8 depicts the flux and rejection of CR and MO dyes with the help of the bare PVDF, PVDF/PVP, and nanocomposite membranes. All the modified PVDF membranes with nanocomposite had higher CR and MO removal rates than the unmodified one. The rejection rate of CR and MO is 95.9% and 93.6% by the PVDF membrane containing 1 wt% HG, while those are 92.9% and 89.5% by the bare PVDF membrane. The difference in dye rejection between CR and MO for each membrane is attributed to the fact that the dyes have different structures and molecular weights.

Additionally, the modified membranes had a negatively charged surface; thus, the repulsion forces between the dye surface and membrane surface charges can be another effective parameter^64,65^. Also, the flux increased by raising HG content in membranes. The flux of CR dye by the M4 membrane containing 0.3 wt% HG was 81.6 L/m^2^ h, which is considerably advanced compared to that by the bare PVDF membrane, i.e., 37.6 L/m^2^ h. Furthermore, the permeation flux of MO dye by M0 and M4 membranes is 39.8 and 85.1 L/m^2^ h, respectively. As a result, PVP-HG addition into the casting solution enhanced dyes' flux and rejection rate.

### Comparison with other blended membranes

Comparing the performance of PVDF-based polymer membranes containing different additives in several studies done with nanocomposite hydrogel membranes in this research is given in Table 4. However, to the best of our knowledge, there was not any study on the modifying PVDF membranes using PVA-GO-NaAlg. As can be seen, the pure water flux, BSA and dye rejection rate, contact angle and flux recovery ratio of the optimal membrane prepared using 0.3 wt% HG compared to other works done in It has been placed in a favorable state and it can be introduced as an efficient material for PVDF membrane modification.

**Table 4:** Comparison of various blended membranes modified of the previously reported literature.

Membrane	Flux (L/m^2^h)	Rejection (%)	Contact angle (°)	Flux recovery ratio (%)	References
PVDF/P-GO-DAA	305	BSA 84	64	71	^66^
PVDF/PVDF-g-PMMA/GO@SiO2	17.5	Direct yellow 99.8 Crystal Violet 99.5	68	–	^67^
PVDF-GO/ZnS	431.9	BSA 87.1	61.7	66.7	^68^
PVDF-P/GO	93	BSA 94	59	78	^69^
PVDF/PVP/GO-PVA-NaAlg	103.2	Conge Red 95.3 Methyl Orange 92.7 BSA 98.5	70.7	90.1	This study

*DAA* d-Tyrosine.

## Conclusion

In this research, PVDF UF membranes were blended with GO-PVA-NaAlg hydrogel as a hydrophilic nanofiller and PVP as a pore-forming agent to enhance their separation and filtration performance in water treatment. The main outcomes are as follows.

- The XRD, FTIR, and FESEM results of the modified membranes demonstrated that the combination of PVA and NaAlg molecules through the hydrogen bonding interactions into GO layers led to the demolition of the well-ordered structure of GO, which proved the successful membrane modification with hydrogel.
- Adding nanocomposite into the membrane increased the hydrophilicity, which reduced the contact angle from 82.5° for the bare PVDF membrane to 65.1° for the nanocomposite membrane containing 1.0wt% HG. The AFM images indicated an increase in the roughness, ranging from 128.9 nm for the bare PVDF membrane to 233.3 nm for the membrane with 0.3 wt% HG.
- The water flux increased from 46.5 to 103.2 L/m^2^h for the optimal nanocomposite membrane containing 0.3 wt% HG and 1.0 wt% PVP, where the water flux increased by about 121%. However, adding more HG content in M5 and M6 membranes generated the membrane pore blockage and decreased the overall porosity and water permeation flux. The GO-PVA-NaAlg nanocomposite improves the rejection rate of BSA, Congo Red, and Methyl Orange by the nanocomposite membranes by 98%, 95%, and 92%, respectively. Furthermore, total and irreversible fouling rates in the optimal membrane decreased by 20% and 72%, respectively.

Due to the improved antifouling characteristics by incorporating hydrogel, the modified PVDF membranes can be employed as a suitable candidate for efficient water purification, particularly for dye removal.

## Data Availability

Data are available [from Armin Ghobadi Moghadam] with the permission of [Alireza Hemmati]. The data that support the findings of this study are available from the corresponding author, [Alireza Hemmati], upon reasonable request.
